# Maximal Glycemic Difference, the Possible Strongest Glycemic Variability Parameter to Predict Mortality in ICU Patients

**DOI:** 10.1155/2020/5071509

**Published:** 2020-08-24

**Authors:** Thanaphruet Issarawattana, Rungsun Bhurayanontachai

**Affiliations:** ^1^Division of Internal Medicine, Faculty of Medicine, Prince of Songkla University, Hat Yai, Songkhla, Thailand; ^2^Critical Care Medicine Unit, Division of Internal Medicine, Faculty of Medicine, Prince of Songkla University, Hat Yai, Songkhla, Thailand

## Abstract

**Background:**

This retrospective study aimed to determine the correlation of blood glucose and glycemic variability with mortality and to identify the strongest glycemic variability parameter for predicting mortality in critically ill patients.

**Methods:**

A total of 528 patients admitted to the medical intensive care unit were included in this study. Blood glucose levels during the first 24 hours of admission were recorded and calculated to determine the glycemic variability. Significant glycemic variability parameters, including the standard deviation, coefficient of variation, maximal blood glucose difference, and J-index, were subsequently compared between intensive care unit survivors and nonsurvivors. A binary logistic regression was performed to identify independent factors associated with mortality. To determine the strongest glycemic variability parameter to predict mortality, the area under the receiver operating characteristic of each glycemic variability parameter was determined, and a pairwise comparison was performed.

**Results:**

Among the 528 patients, 17.8% (96/528) were nonsurvivors. Both survivor and nonsurvivor groups were clinically comparable. However, nonsurvivors had significantly higher median APACHE-II scores (23 [21, 27] vs. 18 [14, 22]; *p* < 0.01) and a higher mechanical ventilator support rate (97.4% vs. 74.9%; *p* < 0.01). The mean blood glucose level and significant glycemic variability parameters were higher in nonsurvivors than in survivors. The maximal blood glucose difference yielded a similar power to the coefficient of variation (*p* = 0.21) but was significantly stronger than the standard deviation (*p* = 0.005) and J-index (*p* = 0.006).

**Conclusions:**

Glycemic variability was independently associated with intensive care unit mortality. Higher glycemic variability was identified in the nonsurvivor group regardless of preexisting diabetes mellitus. The maximal blood glucose difference and coefficient of variation of the blood glucose were the two strongest parameters for predicting intensive care unit mortality in this study.

## 1. Introduction

Acute hyperglycemia or stress-induced hyperglycemia in critically ill patients commonly occurs in the intensive care unit (ICU) [1]. This glycemic alteration is theoretically caused by the stimulation of the counter-regulatory hormones, which primarily respond to inflammation and insulin receptor resistance [1–6]. This abnormal elevation of blood glucose level was essentially related to adverse outcomes in critically ill patients including mortality, acute kidney injury development, nosocomial infection, and peripheral neuropathy [7–13].

Intensive blood glucose control in critically ill patients, to keep the blood glucose level at 80–110 mg/dL by continuous insulin infusion, significantly reduced ICU mortality and morbidity. However, hypoglycemic complication was dramatically high in these studies [14–17]. A NICE SUGAR trial recently demonstrated the appropriated blood glucose level control in critically ill patients at 140–180 mg/dL, resulting in lower occurrence of hypoglycemic complication but without significant difference in ICU mortality [18, 19]. Since then, blood glucose levels have been generally used as a biomarker for glycemic target in the general ICU care worldwide.

Despite of a single blood glucose level, variation of blood glucose level or glycemic variability (GV) during ICU admission was particularly interesting. GV is defined as the magnitude of blood glucose changes during the ICU admission. A high GV might induce oxidative stress similar to hyperglycemia. Several studies have previously found that GV was correlated with ICU and hospital mortality [20–23]. Nevertheless, several GV parameters observed in critically ill patients were considered, such as the standard deviation (SD) of blood glucose, coefficient of variation (CV) of blood glucose, hyperglycemic and hypoglycemic events, blood glucose percentile, and glycemic lability index (GLI) [20, 24–27]. In addition, J-index and maximal blood glucose difference (MGD) were among the common GV parameters in outpatients with diabetes mellitus (DM) [28].

However, there has been no well-established standard GV parameters in the critical care practice. Therefore, this study aimed to determine the correlation of common GV parameters with ICU outcomes and to identify the strongest parameter among the existing GV parameters that correlate with ICU mortality.

## 2. Patients and Methods

### 2.1. Study Design

This is a single-center, retrospective, cohort study to determine the correlation of common GV parameters with ICU outcomes and to identify the strongest parameter among existing GV parameters that correlate with ICU mortality. Electronic medical records of medical ICU patients in Songklanagarind Hospital from June 1, 2014, to December 31, 2015, were reviewed and recorded. The patients' identity including name, surname, and hospital number was concealed. This study was approved by the Ethics Committees of Faculty of Medicine of Prince of Songkhla University with informed consent waiver (EC number: 59-073-14-4) and was retrospectively registered at http://www.clinicaltrials.in.th (identifier TCTR20200515003).

### 2.2. Patients

Adult patients aged ≥18 years who were admitted to the medical ICU for >24 h and had three or more point-of-care (POC) blood glucose samplings during the first 24 h of their ICU stay were initially recruited. We subsequently excluded patients who were readmitted to the ICU within the same admission period, admitted after primary coronary intervention, diagnosed with a hyperglycemic emergency such as diabetic ketoacidosis and hyperosmotic hyperglycemic syndrome, or had a blood glucose level of >500 mg/dL.

All patients underwent the POC blood glucose testing by arterial blood sampling at the beginning of their admission, which was subsequently evaluated every 1–4 h according to the departmental protocol. If the blood glucose level was >180 mg/dL, a protocol continuous intravenous insulin infusion was then started to control the blood glucose level of 100–180 mg/dL. In case of hypoglycemia (blood glucose level, <60 mg/dL) and severe hyperglycemia (blood glucose, >400 mg/dL), the insulin infusion protocol was then aborted and the appropriated urgency management was commenced. All blood glucose levels were evaluated using a standard POC device (ACCU-CHEK Performa®, Roche diagnostic, Thailand).

### 2.3. Data Collection

Patient characteristics such as gender, age, underlying diseases, main cause of ICU admission, APACHE-II score, caloric intake during the first 24 h, number of POC blood glucose sampling in the first 24 h, blood glucose level in the first 24 h, application of insulin therapy, mechanical ventilator support, and ICU outcomes were collected and recorded.

The mean blood glucose level and existing GV parameters including the SD, CV, MGD, and J-index were calculated and recorded. The CV signified the ratio of SD to the mean blood glucose. MGD was defined as the difference between maximal and minimal blood glucose levels. The J-index was calculated by the 0.001 × (mean blood glucose + SD)^2^.

### 2.4. Statistical Analysis

The sample size was calculated by comparing two proportion methods with type I error of 0.05 and statistical power of 80% to detect the mortality difference of 12% between groups [24]; therefore, this study eventually required 498 patients.

All continuous data were expressed as mean and SD or median with interquartile range (IQR) depending on the data distribution. Categorical variables were reported as frequency and percentages. The difference of patient characteristics and all GV parameters between ICU survivor and nonsurvivors was initially compared using the Chi-square, Fisher exact test, independent *t*-test, and Mann–Whitney test as appropriate. Subgroup analysis of ICU outcomes and GV parameters was also performed in patients with and without preexisting DM.

To identify independent factors correlated to ICU mortality, all parameters with the *p* value of <0.1 were selected into the forward, stepwise binary logistic regression model. All GV parameters were tested for multicollinearity before entering. Odds ratio and 95% confidence interval (CI) were then reported as independent factors of ICU mortality.

Subsequently, all interesting GV parameters were then determined based on their power to discriminate ICU mortality according to the area under the receiver operating characteristic (AUROC) curve and were then pairwise compared to the identified strongest GV to predict the ICU mortality.

To evaluate the effect of either blood glucose level control or GV control on ICU mortality, we categorized the patients into four groups according to their mean blood glucose level and MGD. According to the current evidence, a mean blood glucose level greater than 180 mg/dL was classified as poor blood glucose control. In addition, an MGD cut-off value was selected for GV classification based on the current receiver operating characteristic (ROC) and Youden index. Finally, the patients were classified into the following four groups: good blood glucose control/low GV group (group 1), good blood glucose control/high GV group (group 2), poor blood glucose control/low GV group (group 3), and poor blood glucose control/high GV group (group 4). The ICU mortalities of all four groups were then compared pairwise using the Chi-square test.

A *p* value of <0.05 was considered statistically significant. All analyses were computed using MedCalc Statistical Software version 19.2 (MedCalc Software Ltd., Ostend, Belgium).

## 3. Results

### 3.1. Demographic Data and GV Parameters

A total of 1,316 patients were admitted in the medical ICU during the study period. After exclusion, 528 patients were finally included in the analysis. The patients' baseline characteristics are shown in Table 1. Selected patients had a mean age of 62 years, comprising 54.1% males, and 42.6% had underlying hypertension, and respiratory causes were the main causes of ICU admission (65.4%). The median APACHE-II score was 20 (14, 24). About 19.3% of the patients required insulin infusion for glycemic control with the incidence of hypoglycemia of 8%.

The overall mortality in this cohort was 17.8%. Both survivor and nonsurvivor groups were clinically comparable. However, nonsurvivor had significant higher median APACHE-II score (23 (21, 27) vs. 18 (14, 22); *p* < 0.01), higher rate of mechanical ventilator support (97.4% vs. 74.9%; *p* < 0.01), and higher number of median blood glucose sampling (8 (7, 9) vs. 7 (6, 7); *p* < 0.01). No differences were observed in the number of patients receiving insulin therapy, having a DM diagnosis, and number of hypoglycemic events between survivors and nonsurvivors (Table 1).

The mean blood glucose level on the admission day and all existing GV parameters were significantly higher in nonsurvivors (Table 2).

**Figure 1:**
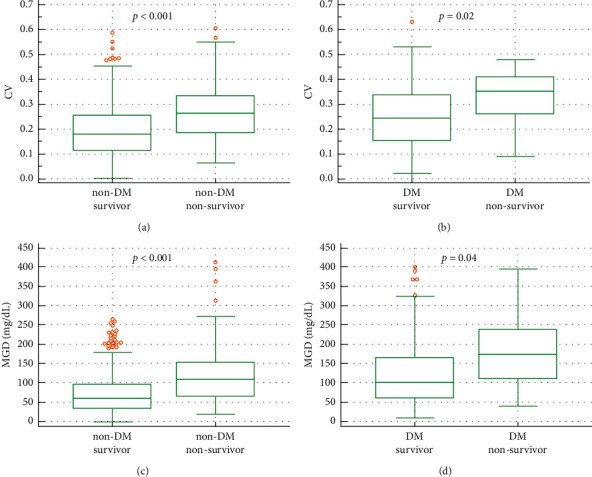
Box plot showing the effect of GV on ICU mortality in patients with DM and non-DM, CV in non-DM (a) and DM (b) and MGD in non-DM (c) and DM (d). Figures were constructed with MedCalc Statistical Software version 19.2.6 (MedCalc Software Ltd., Ostend, Belgium).

In subgroup analysis of preexisting DM, both MGD and CV were found to be significantly higher in the nonsurvivor than that in the survivor group, regardless of the DM status (Figure 1).

### 3.2. Factors Associated with ICU Mortality

From the univariate analysis and multicollinearity test for all GV parameters, age, gender, hypertension, malignancy, causes of ICU admission, APACHE-II score, calorie intake during the first 24 h of ICU admission, presence of insulin therapy, and MGD were then selected into the binary logistic model. Eventually, we found that male gender (odds ratio [OR] 0.49; 95% CI 0.27–0.88; *p* = 0.02), APACHE-II score (OR 1.10; 95% CI 1.04–1.17; *p* < 0.001), presence of insulin administration (OR 2.89; 95% CI 1.16–7.24; *p* = 0.02), and MGD (OR 1.02; 95% CI 1.01–1.03; *p* < 0.001) were among the independent factors correlated to ICU mortality (Table 3).

### 3.3. Discrimination Power of Interesting GV Parameters to Identify ICU Mortality

The AUROC was computed to indicate the power of existing GV parameters to predict the ICU mortality (Table 4). MGD eventually demonstrated the strongest parameter with AUROC of 0.69 (0.64–0.75), followed by CV (AUROC 0.68 [0.62–0.74]), SD (AUROC 0.67 [0.61–0.73]), and J-index (AUROC 0.63 [0.57–0.70]).

To compare the discrimination power between each GV parameter, the pairwise comparison of AUROC was applied. Finally, MGD provided a comparable power to CV (*p* = 0.21) but was significantly stronger than SD (*p* = 0.005) and J-index (*p* = 0.006).

### 3.4. The Correlation of ICU Mortality with the Combination of Blood Glucose Control and GV Control

We selected a mean blood glucose level of 180 mg/dL as the cut-off between good and poor blood glucose control. After performing the ROC analysis and applying the Youden index for MGD to predict the ICU mortality, we selected a GV cut-off value of 83 mg/dL to define low and high GV; this cut-off value had a sensitivity and specificity of 70.8% and 62.4%, respectively.

**Figure 2:**
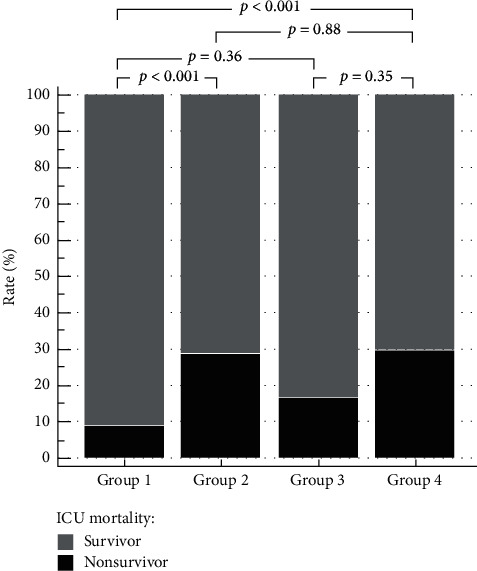
The correlation of ICU mortality and the combination of blood glucose control and GV control, classified into four groups: Group 1 (good blood glucose control/low GV); Group 2 (good blood glucose control/high GV); Group 3 (poor blood glucose control/low GV); Group 4 (poor blood glucose control/high GV). Each group was pairwise compared with the Chi-square test. *p* < 0.05 was defined as statistically significant. Figures were constructed using MedCalc Statistical Software version 19.2.6 (MedCalc Software Ltd., Ostend, Belgium).

We found that group 1 had a significantly lower ICU mortality when compared with groups 2 and 4 (8.9% vs. 28.8% vs. 29.6%, *p* < 0.001). The ICU mortality in group 1 was also lower than that in group 3, but this difference was not statistically significant (8.9% vs. 16.7%, *p* = 0.36). In addition, the ICU mortality rates in groups 2, 3, and 4 were comparable (Figure 2).

## 4. Discussion

In this study, several existing GV parameters, including MGD, CV, SD, and J-index, were related to poor ICU outcomes, particularly mortality. We also found that the nonsurvival group had a higher GV compared to the survival group, regardless of the DM status in critically ill patients. In addition, GV parameters, as well as APACHE-II, were among the independent factors influencing ICU mortality in this study. Among our interesting GV parameters, we eventually discovered that MGD is possibly the strongest parameter related to ICU mortality.

From several studies, acute hyperglycemia in critically ill patients or stress-induced hyperglycemia associated with poor ICU outcomes, including hospital-acquired infection, prolonged ICU stay, prolonged mechanical ventilator days, and higher ICU mortality [10, 29]. Several studies confirmed the finding that tight glycemic control with intravenous insulin infusion resulted in better ICU outcomes. From the recent large clinical study, the optimal blood glucose control level in the critically ill patients between 140 mg/dL and 180 mg/dL has become a standard of ICU care to reduce the mortality and morbidity [18, 29].

Apart from the individual blood glucose level, GV was recently found to be significantly correlated with worse ICU outcomes [24, 30]. Several proposed mechanisms of higher GV and ICU outcomes, including increased oxidative stress, mitochondrial damage, endothelial injury, and coagulopathy, have been identified [31]. Our finding has also supported previous studies regarding the correlation of higher GV and ICU mortality and the observation that it is one of the independent factors related to ICU death.

Egi and Bellomo [25] found that continuous insulin infusion to control blood glucose level could control as well as reduce the GV in critically ill patients and reduced mortality. Unfortunately, our study did not report GV outcomes to control in critically ill patients.

Several GV parameters were applied in the clinical practice in both critically and noncritically ill patients. Several studies supported the application of SD in blood glucose level in critically ill patients [24, 32, 33]. The glycemic gap, defined as the gap of actual blood glucose level at ICU admission and estimated mean blood glucose derived from HbA1C, was also related to worse ICU outcomes [34]. Akirov et al. [23] also found that the third CV tertile had a higher ICU mortality in ICU patients similar with recent studies from India [27, 35]. In addition, the common GV parameter in outpatient setting has been applied in the ICU setting, including J-index, MGD, and mean amplitude of glycemic excursion (MAGE), which also demonstrated the correlation with ICU mortality [21]. However, no study was conducted to identify the strongest GV parameter to predict the mortality in the ICU to date. Donati et al. [36] found that the GLI had the strongest correlation to hospital mortality as compared to SD and CV (AUROC 0.61 [95% CI, 0.58–0.64]), 0.59 (95% CI, 0.56–0.63), and 0.60 (95% CI; 0.58–0.62), resp.). The GLI and other time-based GV parameters are parameters that are involved with the amplitude change of blood glucose over a period of time and required complex calculation [21]. Our study compared several interesting GV parameters and eventually found that the MGD and CV are among the two strongest parameters to predict the ICU mortality compared to SD and J-index. The maximal glycemic difference is defined as the gap between the highest and lowest blood glucose in 24 h, which is easily obtained during serial blood glucose monitoring and does not require the sophisticated formula to be calculated. Therefore, the MGD could be an appropriated and practical GV parameter to monitor the glycemic control and GV in critically ill patients. Although the MGD is the strongest parameter in our finding, the discrimination power derived from AUROC is in the moderate level. Therefore, a larger population study may be required to confirm our findings.

Several studies reporting the benefit of glycemic control and GV control were possibly observed in only non-DM critically ill patients and found that the higher GV in non-DM patients was related to mortality [20, 24, 37]. Prolonged hyperglycemia and adaptation to glycemic excursion have been proposed as possible protective mechanisms of higher GV in critical illness [38]. However, our finding did not support these results. In our study, nonsurvivors had a higher GV regardless of the DM status, which possibly explained the hypothesis of the diabetic paradox in the ICU [39]. The lower incidence of preexisting DM status in our study (24%) compared with previous studies may have influenced this outcome. Caloric supplements and insulin administration may disturb both the blood glucose level and GV [20] but these exogenous factors were comparable between the survival and nonsurvival groups. Therefore, the higher GV in the nonsurvival group in this study could be obtained from endogenous biological effects of stress responses in critical illness.

In the present study, we also demonstrated a possible mortality protective effect of low GV in critically ill patients despite having poor blood glucose control. We found that the group of patients with low MGD had a lower ICU mortality rate compared with the patients with a higher MGD regardless of blood glucose level, similar to a previous retrospective study [40]. These concordant results support the observation that the target of glycemic control in stress hyperglycemia should not only be to optimize the absolute blood glucose level but should also be to reduce GV. However, the appropriated method to control GV has not been definitely established. Thus, further clinical studies are necessary.

The number of blood glucose measurement may influence the GV. The continuous blood glucose monitoring may provide more information about blood glucose variation and glucose complexity in critically ill patients [30]. However, the application of continuous blood glucose monitoring devices in the ICU still required more studies to verify their accuracy and validity [41]. So far, the conventional blood glucose monitoring every 4–6 h could be used instead of continuous monitoring. The median number of blood glucose monitoring in this study was seven times within 24 h, which was more frequent than conventional monitoring; therefore, the calculated GV from conventional monitoring in this study could be reliable. Although our study used the arterial blood glucose level measured by the portable blood glucose monitoring device, a recent study reported that this measurement technique provided a blood glucose level similar to the standard venous blood glucose level [42].

We acknowledged several limitations in this study. First, this is a retrospective study; therefore, missing data were inevitable. However, the main interesting parameters, including blood glucose level, total calories, and insulin administration, were completely extracted from the medical record and the number of blood glucose monitoring was higher than that of conventional monitoring. Furthermore, we excluded patients who had blood glucose examined <3 times during the study period. Therefore, our study results could be applicable. Second, our study focused on the blood glucose level only and GV in the first 24 h instead of during ICU stay, and this GV could reflect the severity of illness rather than specific metabolic dysregulation. However, our results were similar to the recent report from Taiwan that showed that higher GV in the first 24 h of ICU admission was associated with 30-day mortality in septic critically ill patient [43]. Therefore, GV monitoring in critically ill patients may be beneficial. Third, the number of population in our study was essentially lower than several previous studies; however, the main outcomes in this study also yielded similar results, except for outcomes in the preexisting DM status. In addition, our study mainly recruited medical critically ill patients; therefore, our result may not be applied in surgical patients. Several GV parameters were not selected in our study, for example, MAGE or GLI; however, our interesting GV parameters were more popular and easily applicable into the common clinical practice. Finally, our study demonstrated only the correlation between high GV and ICU mortality; thus, lower GV could be a protective factor against mortality in critically ill patients. However, the optimal methods for GV control in ICU settings require further investigation.

## 5. Conclusion

GV was independently associated with ICU mortality. Higher GV was found in the nonsurvivor group regardless of the preexisting DM. MGD and coefficient of blood glucose variation were among the two strongest parameters to predict the ICU mortality in this study. However, the MGD was more practically applied in the clinical practice. The higher MGD within the first 24 h of ICU admission does not only predict ICU outcomes but also stratifies the severity of illness in critically ill patients.

## Figures and Tables

**Table 1:** Demographic data comparison between survivors and nonsurvivors.

	All patients *N* = 538	Survivors *N* = 442	Nonsurvivors *N* = 96	*p* value
Age (median, IQR), years^*∗*^	62 (51,79)	65 (51,79)	66 (51,75)	0.77
Male gender (*n* (%))^†^	291 (54.1)	231 (52.3)	60 (62.5)	0.09
Comorbidities (*n* (%))^†^				
1. Hypertension	229 (42.6)	196 (44.3)	33 (34.4)	0.09
2. Cardiovascular disease	145 (27.0)	121 (27.4)	24 (25.0)	0.73
3. Diabetes mellitus	130 (24.2)	110 (24.9)	20 (20.8)	0.48
4. Renal insufficiency	104 (19.3)	89 (20.1)	15 (15.6)	0.38
5. Neurologic disease	67 (12.5)	59 (13.3)	8 (8.3)	0.24
6. Malignancy	81 (15.1)	59 (13.3)	22 (22.9)	0.02
7. Respiratory disease	69 (12.8)	58 (13.1)	11 (11.5)	0.78
8. Hepatobiliary disease	50 (9.3)	44 (10)	6 (6.2)	0.35
Causes of ICU admission (*n* (%))^†^				
1. Respiratory causes	352 (65.4)	277 (62.7)	75 (78.1)	0.01
2. Sepsis, infection causes	244 (45.4)	192 (43.4)	52 (54.2)	0.07
3. Cardiovascular causes	208 (38.7)	179 (40.5)	29 (30.2)	0.08
4. Neurological causes	85 (15.8)	72 (16.3)	13 (13.5)	0.61
5. Gastrointestinal causes	81 (15.1)	69 (15.6)	12 (12.5)	0.54
6. Renal causes	72 (13.4)	54 (12.2)	18 (18.8)	0.12
APACHE-II (median, IQR)^*∗*^	20 (14, 24)	18 (14, 22)	23 (21, 27)	<0.01
24 h Calorie intake (median, IQR), Kcal^*∗*^	0 (0, 560)	0 (0, 560)	0 (0, 230)	<0.01
Insulin administration (*n* (%))^†^	104 (19.3)	78 (17.6)	26 (27.1)	0.05
Hypoglycemia (*n* (%))^†^	43 (8.0)	31 (7.0)	12 (12.5)	0.11
Number of glucose samples in 24 h (median, IQR)^*∗*^	7 (6,7)	7 (6,7)	8 (7,9)	<0.01
Mechanical ventilator used, *n* (%))^†^	425 (79.0)	331 (74.9)	94 (97.4)	<0.01

^†^Statistical analysis with the Chi-square test; *p* value for significance <0.05. ^∗^Statistical analysis with the Mann–Whitney *U*-test; *p* value for significance <0.05.

**Table 2:** Comparison of GV parameters between survivors and nonsurvivors.

GV parameters	Survivors (*N* = 442)	Nonsurvivors (*N* = 96)	*p* value
Mean blood glucose level	138.8 (118.1, 165.4)	157.9 (129, 182.5)	<0.01
Maximal blood glucose difference (MGD)	66.5 (39.0,114.0)	112 (70.0, 177.0)	<0.01
Standard deviation (SD) of blood glucose	26.2 (15.8, 40.3)	40.3 (25.8, 61.5)	<0.01
Coefficient of variation (CV) of blood glucose	0.2 (0.1, 0.3)	0.3 (0.2, 0.4)	<0.01
J-index	27.1 (19.0, 42.6)	40.0 (23.6, 58.6)	<0.01

All data were described as the median and IQR and analyzed using the Mann–Whitney *U*-test; *p* value for significance, <0.05.

**Table 3:** Independent factors correlated with ICU mortality.

Variables	Odds ratio	95% confidence interval	*p* value
Male	0.49	0.27–0.88	0.02
APACHE-II score	1.10	1.04–1.17	<0.001
Insulin administration, yes	2.89	1.16–7.24	0.02
Maximal blood glucose difference (MGD)	1.02	1.01–1.03	<0.001

**Table 4:** AUC of each glycemic variability parameter to predict ICU mortality.

Glycemic variability	Area under the curve (AUROC)	95% CI	*p* value^*∗*^
Maximal blood glucose difference	0.69	0.64–0.75	—
Coefficient of variation	0.68	0.62–0.74	0.21
Standard deviation	0.67	0.61–0.73	0.005
J-index	0.63	0.57–0.70	0.006

^*∗*^Pairwise comparison of the ROC curve of each glycemic variability parameter compared with the maximal blood glucose difference (MGD).

## Data Availability

The datasets used and/or analyzed during the current study are available from the corresponding author on reasonable request.

## Abbreviations

SD: Standard deviation
CV: Coefficient of variation
GLI: Glycemic liability index
MGD: Maximal blood glucose difference
DM: Diabetes mellitus.
